# Who Is Able to Resist What Is Forbidden?—The Relationship between Health Literacy and Risk Behaviours in Secondary School Students in the Broader Social and Educational Context

**DOI:** 10.3390/ijerph19159381

**Published:** 2022-07-31

**Authors:** Dorota Kleszczewska, Joanna Mazur, Katarzyna Porwit, Anna Kowalewska

**Affiliations:** 1 Institute of Mother and Child Foundation, 01-211 Warsaw, Poland; 2 Department of Humanization in Medicine and Sexology, Collegium Medicum, University of Zielona Gora, 65-729 Zielona Gora, Poland; joanna.mazur@hbsc.org; 3 Centre of Migration Research, University of Warsaw, 02-093 Warsaw, Poland; katarzyna.porwit@uw.edu.pl; 4 Department of Biomedical Aspects of Development and Sexology, Faculty of Education, Warsaw University, 00-561 Warsaw, Poland; a.kowalewska@uw.edu.pl

**Keywords:** education track, gender differences, health literacy, neighbourhood deprivation, risk behaviours

## Abstract

In the last Health Behaviour in School-Aged Children (HBSC) survey conducted in Poland in 2018, a group of 17-year-old adolescents (*n* = 1663; mean age 17.63 ± 0.36 years) was included outside the international protocol. This allowed an assessment to be made of their level of health literacy (HL) using the 10-point HBSC research tool. The aim of the study was to investigate the relationship between HL and risk behaviours (RB). A standardised index of RB in the last 30 days was considered as an outcome measure. This index was significantly higher in the group with low HL (0.318 ± 1.269) in comparison with the group with high HL (−0.083 ± 0.962). In a multivariate linear regression model, the strongest predictors of RB were gender, academic performance and level of regional deprivation, but the association with HL remained significant. This significant association persisted in general schools and in girls but disappeared in vocational schools and in boys. It was also shown that in rural areas, good academic performance has a less significant impact on RB if the HL level is low. The analyses led to the conclusion that when examining the relationship between HL and RB in older adolescents, it is advisable to take into account gender, the educational track and neighbourhood characteristics.

## 1. Introduction

In view of the increasing inequalities in health that apply to all demographic and social groups, including young people [1], it is advisable to look for factors that may be able to narrow these inequalities. One of these factors could be health literacy (HL), which is, according to the World Health Organization’s definition, understood as ‘the cognitive and social skills which determine the motivation and ability of individuals to gain access to, understand and use information in ways which promote and maintain good health’ [2] (p. 4). Despite the fact that the term was used for the first time in 1970 [3], it is mainly in the past decade that an increasing interest in HL could be observed. This is probably driven by the proven evidence of the relationships between HL and its outcomes, which have many aspects.

According to a broad systematic review by Broder et al., in which 12 definitions and 21 models of HL have been analysed, HL in children and young people is described as comprising variable sets of key dimensions, appearing as related abilities, skills and knowledge that enable a person to approach health information competently and effectively and to derive health-promoting decisions and actions. As a result of this research, the authors claimed that children and young people are expected to develop skills today that influence their health (outcomes) and well-being over their life course and reduce health expenditures. Broder et al. concluded that young people are at a crucial stage of development, characterised by many physical, emotional and cognitive changes [4]. That is why they can be tempted by activities that are not appropriate for their age.

The association between low HL and unhealthy behaviours mentioned by Broder et al. [5] is especially important to understand and dangerous with regard to young people [6]. This is primarily because adolescence can be defined as a difficult time, a period of ‘teenage rebellion’ when young people contest widely accepted norms on principle and experiment with the forbidden [7]. The literature calls these ‘forbidden activities’ risk behaviours (RB), and among them lists smoking, drinking, early sexual activity and using marijuana [8]. The unfavourable effect that these activities have on the health and well-being of adolescents has been proved numerous times, but young people still engage in various RB, and this is an increasing trend [9]. Analysing the factors that either drive adolescents into RB or inhibit those behaviours is still relevant [10]. HL may be an effective way to motivate the younger part of society to undertake health-oriented efforts. It is particularly important because young people still have the possibility to develop good health-related habits that may result in better health in the future.

If we consider inequalities in health, the neighbourhood must be taken into account. The environment—the neighbourhood or place of residence [11,12], school [13,14] and family [15]—where a young person grows up is crucial for their health and for the behaviour which influences their health. Poorly supportive neighbourhood circumstances result in young people living an inferior lifestyle. Children from less affluent environments spend more time watching TV [16] or using a computer [17], eat less healthily [18,19], and are more likely to reach for stimulants [20].

The strong positive correlation between HL and both family affluence and the social and structural aspects of the environment in which school-aged children grow up was confirmed by the authors of this article in previous studies [21] that covered 11,521 students aged 13–15 in three Central and Eastern European countries (Slovakia, Czech Republic and Poland). This correlation was also shown in Lithuanian research, in which it was demonstrated that the factor with the greatest impact on raising HL levels in young people is school [22].

In the light of these data, it is reasonable to analyse the school environment more precisely by taking into consideration the type of school that a young person attends. In the literature, there are studies presenting the differences in HL levels between adolescents in general schools and those in vocational schools. Danish research has shown that young people from vocational schools come from less privileged backgrounds and that low HL is associated with unhealthy behaviours in this population [23]. Further research into this matter is, therefore, absolutely justified.

This paper extends previous studies based on data from the Health Behaviour in School-Aged Children (HBSC) network, which deal with the RB syndrome [24], the level of HL and selected social determinants [25] and, more generally, the relationship between the place of residence and health-related outcomes [26,27,28].

HBSC is a 30-year-old network of researchers from 50 countries in Europe and from Canada who organise a questionnaire study on the health behaviours of 200,000 adolescents aged 11, 13, and 15 every four years, based on the same international protocol.

In 2020, a national report was issued in Poland [29] comparing a range of health indicators in older adolescents and using the same data source (HBSC 2018). Seventeen-year-old adolescents were examined as part of the study group, with the type of school included as an important factor. The primary focus was on gender differences, changes between the ages of 11 and 17 and, in older adolescents, the relationship with school type. However, the report did not present data on HL, did not include composite behavioural indices and did not analyse social determinants.

The results of the systematic review by Fleary et al. suggest that there is a meaningful relationship between health literacy and adolescent health behaviours. To fully understand the role of health literacy in health-related decision-making among adolescents, future research should use comprehensive definitions and measures of health literacy and integrate theoretical frameworks for health behaviour and adolescent development in the study design. Somewhat in response to this conclusion, it seems that this paper, which takes the RB syndrome as the main outcome, fills a knowledge gap and provides a basis for identifying directions for further research. The authors of the aforementioned research believed that developing validated and objective measures of health literacy that assess all of its aspects (functional, communicative/interactive, critical and media health literacy) and integrating developmental theory in research design is critical for determining the relationship between adolescent health literacy and health behaviours, as well as identifying areas for intervention. Intervening in health literacy may likely improve health behaviours in adolescents, as it provides the tools for converting knowledge into behaviour and empowers adolescents in health-related decision-making now and throughout their lives [30].

The authors of this paper follow this scope and would like this research to be in line with Fleary’s conclusion. The authors claim that the level of health literacy in children of secondary school age has an impact on their health-related behaviours—in this case, the RB—and that the strength of this relationship depends on their educational track and the neighbourhood in which they live.

To confirm this, the following research questions were asked:

- Is there a significant correlation between a high level of HL in boys and girls attending secondary schools and a reduction in their RB?
- Does the relationship between HL and RB remain significant after controlling for school factors related to educational track and academic performance?
- To what extent do factors related to neighbourhood deprivation and level of urbanisation change the strength and direction of the relationship under examination?

## 2. Materials and Methods

### 2.1. Respondents

The Polish education system has undergone several changes during the past 25 years. From 1999 to 2019, adolescents who finished primary school (aged 13–16) attended lower-secondary schools, called gymnasiums, followed by upper-secondary schools. The gymnasiums have been gradually phased out, and, currently, education is divided into two instead of three tiers (primary vs. post-primary). Primary school education lasts 8 years, and after graduating, adolescents typically continue it at a secondary school, which takes 3 or 5 years to finish. After graduating from general secondary school or technical secondary school and passing the final leaving exam, called ‘Matura’, one can pursue higher education at university level. In secondary school, Polish youth can choose a general or vocational track. Vocational education is mainly provided by technical secondary schools, as the alternative sectorial vocational schools exist at the first and second stages, where pupils receive work experience and qualifications through apprenticeships.

Data included in this study come from 2018/2019, when upper-secondary schools were still present in the Polish educational system during the transition period, and the youth who took part in this survey were still attending this ‘old’ tier of schooling, having had completed a gymnasium 1–2 years before. The study included 1663 secondary school students who were included in the Polish study outside the international protocol for the latest round of HBSC surveys conducted in 2017/2018. These students completed the same questionnaire as the group of 15-year-olds routinely participating in HBSC surveys according to the international protocol. The structure of the extended study was identical and followed the same protocol [31]. A total of 1663 students with no missing data for the key indices (RB and HL) were eligible for analysis. The majority (93.2%) were surveyed in 2018. The mean age of the respondents was 17.63 years (SD = 0.36), with an age range of 16.5 to 18.5 years. The sample included 56.0% general secondary school students and 44.0% students of technical and other vocational schools. The respondents were predominantly girls (53.5%). The proportion of girls from general schools was also significantly higher than the proportion of girls from vocational schools (59.2% vs. 46.1%; *p* < 0.001). Data were drawn from 84 school classes and 55 schools located in 51 provinces (16 voivodships), whereas the number of respondents ranged from 12 to 59 per school. The HBSC 2017/2018 sampling design included stratification according to the local deprivation index. This means that schools from regions with different levels of economic development were reached. The deprivation index is a composite indicator that measures a region’s level of development based on external data. It has been defined as a combination of subindices for population income, employment, living conditions, education and access to goods and services [32]. The proportions of students from districts classified into successive quintiles of deprivation, in which Q1 denotes the poorest regions, were, respectively: Q1 9.6%, Q2 18.9%, Q3 16.1%, Q4 17.4% and Q5 38.0%. In the study sample of 17-year-olds, 41.2% lived in rural areas, and 58.8% lived in urban areas, of which 21.4% lived in cities with a population of more than 100,000 inhabitants.

The survey was conducted on school premises, using a traditional paper questionnaire. The questionnaire for the study and its organisation, including the procedure for obtaining the consent of parents of under-age students, received a positive opinion from the local bioethics committee operating at the Institute of Mother and Child in Warsaw (17/2017 on 30 March 2017).

### 2.2. Dependent Variable

The main dependent variable is the Risk Behaviour Index (RBI), which was constructed from questions about RB in the past 30 days. For the purpose of this study, the following were considered: smoking traditional cigarettes, smoking e-cigarettes, drinking alcohol, getting drunk and using marijuana. The authors of the present study called them risk behaviours to stay in line with the main HBSC research protocol and the name of the tool used to measure these unhealthy activities. This approach has been applied by Bulgarian researchers before [33]. For the purpose of this study, the index (RBI) was constructed from four questions (excluding the question about drinking alcohol). The response range for each of these questions was reduced so that the responses fell into five categories. For frequency of drinking alcohol, this was the original version of the question that the HBSC research network has been using for years: never, 1 time, 2–3 times, 4–10 times and more than 10 times. The questions on the frequency of smoking, using e-cigarettes and using marijuana were adapted for the international HBSC survey protocol from the ESPAD (European School Survey Project on Alcohol and Other Drugs) project [34,35]. The original seven response categories were reduced to five: never, 1–2 days, 3–5 days, 6–19 days and 20 days or more. A summary index was created using the principal component analysis (PCA) regression method. This is a standardised z-score scale with a population mean of 0 and SD = 1. Positive values indicate RB at a frequency higher than the population mean. In the sample of Polish 17-year-olds, the scale was homogeneous, with the main component explaining 54.43% of the total variability. Cronbach’s reliability coefficient was 0.672. Removing any one of the questions does not improve the reliability of this scale.

### 2.3. Independent Variables

The first group of factors concerned personal competencies that may influence RB. The scale of HL (HLSAC—health literacy in school-aged children) was taken into account; this was created on the basis of the Finnish prototype and was included in the HBSC 2017/18 research protocol following validation studies that were also conducted in Poland [36]. The instrument consists of ten items, two items for each of five predetermined theoretical components: theoretical knowledge, practical knowledge, critical thinking, self-awareness and citizenship. The respondents were asked to rate the extent to which they agreed with the statements by selecting one of four responses ranging from ‘not at all true’ to ‘absolutely true’ [37]. According to the guidelines provided by the authors of the scale, the summary index has a range of 10 to 40 points, and the higher the score, the more favourable the level of HL. It was also assumed in previous studies that the categories of a low, an average and a high level of HLSAC are determined according to point ranges of 10–25, 26–35 and 36–40 points, respectively [25]. In the study sample of Polish 17-year-olds, the HLSAC scale was homogeneous, with the principal component explaining 43.63% of the joint variability. Cronbach’s reliability coefficient was 0.856. The mean HLSAC index was 31.07 (SD = 4.46) in this group of respondents.

Subjective assessment of academic achievement was also included in the group of personal competencies. Students were asked to rate their academic achievement against other students in the class on a visual scale, adopting the graphic image of a ladder according to McArthur’s concept [38]. There were 11 possible ratings, ranging from 0 (worst academic performance) to 10 (best academic performance). The evaluation of a student’s position in the classroom ranking on a vertical visual scale was first used by E. Goodman [39]. The following ranges are conventionally used for poor, average and good academic performance: 0–4, 5–7 and 8–10 points, respectively. Earlier studies have shown that in the middle school student population, this type of scale correlates with objective scores on state tests [40]. The secondary school students achieved an average score of 5.86 (SD = 2.07).

The second group of factors included sociodemographic variables. Family wealth was examined with the revised Family Affluence Scale (FAS III), which is a tool used to measure the material status of the families of students surveyed in HBSC studies [41]. FAS III includes six items, namely the number of family cars, number of computers in the family, having a bedroom of one’s own, number of bathrooms, having a dishwasher in the household and number of family holidays in the past year [42]. Its validity has been assessed in quantitative and qualitative studies, which have also been conducted in Poland [43]. The scale ranges from 0 to 13 points. Low, average and high level of wealth is determined by the ranges of 0–6, 7–9 and 10–13 points, respectively. The FAS scale in the study sample of 17-year-olds had a univariate structure and, despite its rather poor reliability (Cronbach’s alpha was 0.539 in this case), it is a tool recommended by the HBSC research network for analyses of social inequalities in health [41].

In the group of sociodemographic factors, the following were also considered: gender (male, female), type of school (general school, vocational school), deprivation index quintile and place of living (urban, rural).

The characteristics of the sample, together with the mean standardised RB indices, can be found in Table 1.

### 2.4. Statistical Analysis

A psychometric analysis was performed of the scales used, testing their homogeneity with the PCA method and their reliability with Cronbach’s coefficient. The differences between the indices in groups distinguished by socioeconomic characteristics and personal competencies were compared using the nonparametric Mann–Whitney test (two groups) or the Kruskal–Wallis test (more than two groups). The chi-square test for categorised variables and Spearman’s rho coefficient for continuous variables were used to examine the relationship between the variables under study. In the multivariate analysis of the determinants of the variability of the RBI, a classical linear regression, as well as a general linear model (GLM), were used. Diagnostics of the linear regression model included R-squared coefficient of determination, F test of ANOVA and variance inflation factor (VIF) analysis. A series of two-way and three-way interactions were tested in order to determine the best GLM model. The presentation of the GLM results is provided in the Supplementary Materials, and marginal means from this model are shown on the graph.

## 3. Results

### 3.1. Prevalence of Risk Behaviours

In the study group of 1663 second-year secondary school students, the percentage of traditional cigarette smokers was slightly higher than that of electronic cigarette smokers (32.5% vs. 28.7%). The difference to the disadvantage of boys was statistically significant only in the second case. There were more boys than girls who smoked both types of cigarettes, but the difference was more significant for e-cigarettes. Three-quarters of the respondents had drunk alcohol at least once in the previous month, and every fourth respondent had gotten drunk at least once during that time. Gender-dependent differences to the disadvantage of boys were found only with respect to the frequency of alcohol abuse. Almost every fifth boy and every tenth girl had had contact with marijuana in the previous month (Table 2).

### 3.2. HL and RBI Level According to Sociodemographic Factors

Table 1 presents the mean values of the RBI according to HLSAC level, school performance and six sociodemographic characteristics. The prevalence of RB decreased significantly with improvements in HL (*p* < 0.001) as well as with improvements in school performance. The RB were found to be significantly more frequent among boys and in areas with higher levels of deprivation. The association between place of residence and family wealth was on the verge of statistical significance, with a tendency towards worse results in cities and in wealthier families.

The sociodemographic determinants of HL levels were also demonstrated (Table A1 in Appendix A). The correlation between gender and family wealth appeared weaker than with the remaining variables. In contrast, the correlation between neighbourhood deprivation level and academic achievement was the strongest. HLSAC levels were significantly lower among residents of poorer provinces and among poorer students. Relatively better HL was observed in residents of urban areas (compared with rural areas) and by students from general secondary schools (compared with those from vocational secondary schools).

### 3.3. Correlation between HLSAC and RBI

The correlation coefficient between HL and RBI was equal to −0.091 across the entire study group, and the strength of the relationship was weaker in vocational schools than in general secondary schools. Gender appeared to be a moderating factor in this relationship. In both types of schools, a significant negative correlation was found only for girls and was at the same level (rho = −0.126) in both cases. No significant relationship was shown for boys in either type of school. The stronger correlation in general secondary schools may, therefore, be due to the higher representation of girls among these students (Table 3).

### 3.4. Multivariate Analysis

Table 4 presents the estimation of a multivariate linear regression model.

School performance, gender, deprivation, FAS, HLSAC and place of residence were introduced to the final model as significant predictors of RBI variability. Only school type was found to be insignificant (*p* = 0.144). However, school type moderates the strength of the relationships under examination, as was confirmed by the two models estimated for students attending general (GE) and vocational (VT) schools. Notable differences were related to the association of the RBI with HLSAC and with environmental variables. In general schools, the impact of the level of deprivation in the region was not significant. In vocational schools, the association with place of residence was not significant, whereas deprivation was one of the most important determinants of RBI variability. The association with HLSAC became apparent only in GE schools (Beta = −0.075; *p* = 0.026), while it was insignificant in VT schools (Beta= −0.052; *p* = 0.174).

The respective gender-specific models also differed considerably (Table 5). School type does not affect variation in RBI in either boys or girls. The negative association between RBI variability and HLSAC was found to be much stronger in girls (Beta = −0.084; *p* = 0.015) than in boys (Beta = −0.044; *p* = 0.237). In boys, only a negative relationship with academic achievement (*p* = 0.004) was evident, meaning that good school performance protects against smoking, alcohol drinking and cannabis use. In girls, similar to the previously discussed model for the total sample, all the analysed factors proved significant. In addition to high HL, less frequent participation in risk behaviours co-occurred with: living in a rural area, higher neighbourhood affluence and better school performance. Only the relationship with family affluence as measured with the FAS scale was positive, which means that the girls from more affluent families were more prone to RB.

**Figure 1:**
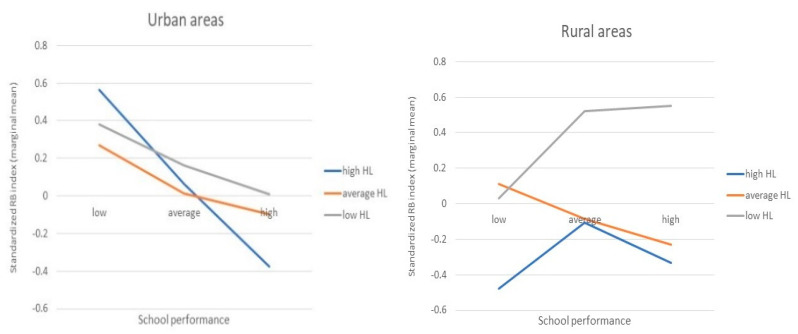
Graphical representation of the interaction between place of residence, health literacy (HL) level and academic performance as predictors of risk behaviour (RBI) index variability.

Additionally, a series of multivariate GLM models were estimated to find independent predictors of variation in the RBI and to examine the interaction between those predictors. This time, we looked for significant interactions with academic achievement, which has proven to be the most significant predictor in previous analyses (with the highest beta). The results are presented in Table S1. In model 1, in which only the main effects are included, six factors were significant. The significant but weaker association between place of residence and HLSAC level persisted. When the three-way interaction was introduced, the main effect of the place of residence and school performance was no longer significant. The association with the HLSAC level is expressed by both the main effect and the interaction with place of residence and school performance. A graphical representation of the interactions in question is shown in Figure 1. These are the so-called marginal means from model 2. In urban areas, as academic achievement improves, the value of the RBI at each level of health competence decreases. Among very good students, a steep decline in RBI becomes apparent as health competence improves. High health competence does not protect against RB if the student has poor academic achievement. The shape of the examined relationship changes significantly among rural residents in the two extreme HLSAC groups. Good students with low levels of HL are very likely to display RB. Therefore, the difference between the three HL levels in the group of good students is even greater than it is among urban residents. In contrast, the protective effect of high HL is more pronounced in the group of poor students than it is in the cities.

All the models confirmed a significant relationship between RB and health literacy as well as with several educational and neighbourhood factors. However, they all explained the variation in the RBI analysed to a small extent, which peaked at around 9% for girls. Collinearity was not detected in the VIF analysis. In the model estimated for the total sample (Table 4), the maximum VIF was equal to 1.329, with a minimum tolerance of 0.753.

## 4. Discussion

### 4.1. Main Findings in Relation to the Hypothesis

The article presents data on 1663 students from the sophomore year of upper-secondary schools in Poland, according to the education system that was in place in Poland in the school year 2017/2018. These students were surveyed according to the HBSC study protocol, which was being carried out simultaneously, although the international protocol covered only the lower grades and lower tiers of education. The focus of the study was the exacerbation of RB associated with substance abuse, as well as HL levels. The issues examined included the relationship between HL and RB adjusted for a range of social factors, academic success and the social determinants of RB and HL.

A standardised index of four RB (drinking alcohol, using marijuana, smoking traditional cigarettes, smoking e-cigarettes) was used as the main outcome measure. Previous publications from HBSC studies confirmed the validity of combining RB into a single factor together with sexual behaviour. To date, however, the use of e-cigarettes has not been included in the composite indices [44].

The results of this study confirm the authors’ hypothesis that there is a significant association between the high level of health literacy in children of secondary school age and their less frequent RB. Furthermore, this relationship depends on the demographic and socioeconomic circumstances in which the secondary school students are growing up.

The following discussion refers to the research questions and also explores gender differences more in-depth.

### 4.2. Discussing Research Questions

#### 4.2.1. The RB of Secondary School Students

In order to discuss the answer to the first research question, an analysis of the prevalence of RB among the youth needs to be performed. In the present study, the boys performed worse, but this was mainly influenced by the large differences in the answers for getting drunk (37.9% vs. 32.3%) and marijuana use (19.2% vs. 11.3%). As far as the remaining analysed behaviours are concerned such as smoking traditional cigarettes (33.5% vs. 31.3%) or drinking alcohol (74.4% vs. 72.8%), the girls had higher scores. This is quite surprising, as we are accustomed to finding that it is men, not women, who are more prone to engaging in RB. This negative trend has also been observed among young adults to the disadvantage of women [45]. These findings may suggest a dangerous, increasing trend for girls and women drinking, smoking and using drugs more often than men.

There is a different situation regarding the issue of smoking electronic cigarettes and gender distribution in this relationship. The results are unfavourable for the boys examined in the present study while being in line with the research of Patanavanich et al. [46]. These authors also proved in their study that boys reach for e-cigarettes more often than girls. They examined 6238 students from 22 schools, with a mean age of 12.9 years (range: 11–16 years). In their study, the prevalence of ever having used e-cigarettes among the boys was significantly higher than among the girls (11.2 vs. 2.8%). Other factors analysed by a Thai researcher also confirm the results obtained in this study: students from more developed areas displayed the highest frequency of e-smoking. Students with worse school performance, measured with the grade point average (GPA), were more likely to report having ever used e-cigarettes than students with a higher GPA [46].

#### 4.2.2. The Relationship between HL and RB

We turn next to aspects that exacerbate RB among adolescents. The aim of the study was to analyse the relationship between RB and HL as a possible factor that could inhibit such behaviours. This is in line with previous international studies [47,48,49].

To answer the second research question, the authors of this paper analysed several determinants of HL in adolescence. The level of HL of the youths in the study depended on environmental factors and demographic determinants. It was proved that the level of HL depends on gender—the girls scored higher on HL, but only in the average category (71.1 vs. 75.7), whereas at the high level of HL, the boys scored better (17.8 vs. 16.2). The HL level was higher among young people from more affluent backgrounds and those with better school achievements. This is in line with previous international studies on this subject. The authors of an Indonesian study led by Prihanto [50] achieved similar findings and identified an association between health behaviour and both HL level and socioeconomic factors among secondary school students in Surabaya, Indonesia. A cross-sectional study was conducted, with 1066 students evaluated as respondents. The results of multivariate analyses showed that better HL levels were driven by social and economic factors and that this relationship was associated with better health behaviour. The factors mentioned were: being female, having better academic performance, having better grades and the father’s education being higher. The factor that showed the strongest association with better health behaviours was gender. In this research, Indonesian girls were less likely to smoke or use drugs than boys. Quite similar findings for gender were presented by Finnish researchers who analysed the co-use of alcohol, tobacco and other drugs among Finnish undergraduates. They used regression analysis to assess the associations between slightly different sociodemographic and lifestyle characteristics and alcohol and drug use. The data were collected through an online questionnaire at the University of Turku, Finland (1177 students). The demographic characteristics significant for alcohol and drug use included: being male, being single, not living with parents during term and, to some extent, religious beliefs. Age, depressive symptoms, perceived stress, self-rated health, health awareness, income sufficiency and academic variables were not associated with the RB mentioned [51].

#### 4.2.3. School Type and School Performance Are Significant Factors in the Relationship between HL and RB

As regards school and school performance, this study confirms that there is an association between better grades and less frequent RB. It could be concluded that students who pay more attention to education are less likely to undertake risk behaviours. Such a conclusion could be logical, but it should also be stressed that this relationship may be inverse—children who engage in risk behaviours more frequently have worse grades at school. What can be said with confidence when it comes to discussing the results of this research is that there is a significant correlation between those two factors, and further analyses to understand the causes of this association need to be undertaken.

Furthermore, the analyses strongly emphasise academic achievement as an important factor with the potential to modify the relationship between HL and RB. Lithuanian studies have also revealed higher HL levels among students with better grades. These findings are consistent with the outcomes of previous research, indicating that academic achievement is a predictive variable for HL in young people [22,52]. Academic achievement and the educational aspirations of young people may be determined by numerous factors, including the family’s cultural capital and the influence of the neighbourhood [53].

#### 4.2.4. Other Socioeconomic Determinants of the Relationship between HL and RB

However, in the 17-year-old group, the type of school has been confirmed as a factor differentiating HL levels, with students attending general secondary schools performing better. This might indicate that there are not only age-related changes but also sociocultural factors. Students aged 13–15 attended secondary schools, which was the compulsory secondary tier of education in Poland at the time, and represented a complete cross-section of the society. According to the same research conducted in 2018 [40], the percentage of students from wealthier families and with a higher subjectively assessed social status was higher in general secondary schools than in vocational schools. In the tested group of 17-year-olds, 9.5% presented low HL levels. When it comes to the HBSC findings concerning 15-year-olds, this percentage ranged from 6.0% in Macedonia to 17.7% in the Czech Republic [25].

Many authors emphasise that social inequality analyses need to take into consideration a complex set of correlations. An independent study was conducted on 2223 students from Polish secondary schools using the Health Literacy Survey Questionnaire tool, which consists of 47 items (HLS-EU-Q16) and investigates approximately 10 social factors. A multipoint analysis confirmed only the connection with the parent’s level of education, the self-assessed economic situation and the city size. Interestingly, differences between the individual school types were not confirmed, even in the univariate analysis. However, the research was conducted only on a regional scale and included 20 schools [54]. Our research examined whether there was any interaction between HL levels and other factors that affect the RBI. The outcome was that the primary result of HL remains significant in the model that includes interactions, but there is also a significant interaction between HL, academic achievement and place of residence as predictors of RB. When the interactions are included in the model, the primary effect of the latter two disappears. At the same time, a relationship with family affluence that was not revealed by simple univariate analyses becomes apparent.

Numerous studies indicate that the educational system is a factor in deepening social inequality. According to research conducted in Austria, Belgium, Germany and the Netherlands, students attending vocational schools are more likely to engage in RB [55]. Longitudinal studies conducted in France also indicated that a young person displaying more beneficial health behaviour is more likely to choose a path of education that grants them the opportunity to attend university [56].

#### 4.2.5. Importance of the Neighbourhood’s Influence on the Relationship between HL and RB in Adolescents

This paper also focuses on the neighbourhood as one of the main factors that affect adolescent health behaviour. In an attempt to find the proper answer to the third research question, we could conclude that the place of residence does matter. The findings from this study confirm that students from less privileged backgrounds engage in RB more often than their colleagues from more affluent neighbourhoods. These results are in line with previous analyses showing that the environment in which young people are raised and grow up has an impact on their behaviour. A negative correlation between poor living circumstances and RBI was observed in the case of young people from backgrounds with the highest levels of deprivation and those from the least affluent families.

Simple analyses indicate a significant correlation between intensified RB and the regional deprivation index, a weaker tendency for higher RB in cities as compared with rural areas and a lack of correlation with family affluence level according to the FAS scale. The correlation between these factors and HL levels was stronger. Higher HL levels were observed in cities and in students from wealthier families. According to Aljassim’s and Ostini’s systematic review, differences in HL between residents of cities and those of rural areas may become less pronounced when other social status metrics are taken into consideration [57]. Theoretical models and findings from numerous empirical studies indicate that there is value in evaluating independent health gradients associated with the level of individual and neighbourhood deprivation [58]. The social and economic status of the neighbourhood, the status of the family and the quality of schooling have previously been examined as significant factors in the frequency of adolescent RB [59]. As an example, it is worth mentioning the study led by Gerra based on data from a very well-known international study called the European School Survey Project on Alcohol and Other Drugs (ESPAD). In 2015, 15- to 16-year-old students from 28 countries were examined. The study showed that cannabis use among adolescents was significantly associated with a lower socioeconomic background [60].

Our research also indicates a highly statistically significant correlation between HL levels and regional deprivation, but the observed linear relationship was not as strong as in the case of the RBI.

The findings allow us to evaluate the manner in which HL changes by the end of the second decade of a person’s life. Furthermore, we can talk about the systematic increase in HL that can be observed between the 13th and the 17th year of life.

### 4.3. Strengths and Weaknesses of the Study

The limitations of this study should also be noted. Its cross-sectional nature does not allow us to establish a causal relationship between the propensity for RB and its potential correlates, including HL level. The models presented explain the variation in the RBI only to a small extent. However, creating a more complete model was not our intention. We aimed to explore the association between HLSAC and RB in a robust way after adjusting the analyses for several sociodemographic factors. Whereas almost all the variables were derived from self-reported data, information on neighbourhood wealth was obtained from an independent source. The added value of the analyses is their relevance to publications from HBSC surveys based on data for younger students collected at the same time and according to the same procedure. It was, therefore, possible to assess whether HLSAC is a good alternative to other survey tools for older adolescents [54] and to compare changes in HL in the second decade of life. The advantage of the study is the large nationally representative sample of 17-year-olds and the fact that analyses were conducted separately for different types of schools and separately for boys and girls. The dominant finding with practical implications is the very strong association between RB and academic achievement. Educational achievement was also found to be a modifier of the association between HLSAC and RB, which was more pronounced in rural areas. This points to the practical implications of the results, including the need for interventions to increase HL among rural students with good academic performance but deficits in HL.

## 5. Conclusions

With reference to the research questions posed at the beginning, it can be stated that a higher level of health literacy plays a protective role against RB undertaken by young people, and it could be a means of inhibiting selected RB. The association between health literacy and RB is much stronger for girls than it is for boys at about the age of 17 and remains significant after controlling for other educational and social factors. For both the overall sample and the subgroups distinguished by gender, good academic achievement is the strongest predictor of RB. Adolescents from general and vocational schools show similar levels for the RB analysed. School type can only be considered a factor that modifies the determinants of RB. Compared with general schools, the neighbourhood characteristics in vocational schools are more significant as predictors of RB. The place of residence makes little difference in the frequency of engaging in RB. However, in young people from rural areas, the protective effect of good academic achievements is reduced if those achievements are not accompanied by a high level of health literacy. It is important to take the social and economic determinants into consideration when planning intervention programmes dedicated to young people, especially considering the aspects of the neighbourhood in which they are growing up.

## Figures and Tables

**Table 1:** Sample characteristics and standardised index of RB according to sociodemographic characteristics of adolescents aged 17.

	*n* (%)	RB (M ± SD)	*p*
Gender			
Male	774 (46.5)	0.148 ± 1.151	<0.001
Female	889 (53.5)	−0.128 ± 0.828	
Type of school			
General school (GE)	932 (56.0)	−0.086 ± 0.898	0.005
Vocational school (VT)	731 (44.0)	0.111 ± 1.109	
Place of living			
Urban	975 (58.8)	0.035 ± 0.992	0.047
Rural	682 (41.2)	−0.048 ± 1.013	
Quintiles of neighbourhood deprivation			
Q1—high deprivation	160 (9.6)	0.146 ± 1.185	0.001
Q2	315 (18.9)	0.159 ± 1.090	
Q3	267 (16.1)	0.081 ± 1.072	
Q4	289 (17.4)	−0.061 ± 0.929	
Q5—low deprivation	632 (38.0)	−0.120 ± 0.881	
Family affluence scale (FAS)			
Low	481 (29.3)	−0.048 ± 0.963	0.102
Average	805 (49.0)	0.005 ± 1.019	
High	357 (21.7)	0.053 ± 1.002	
School performance			
Low	381 (23.0)	0.212 ± 1.149	<0.001
Average	918 (55.4)	−0.019 ± 0.966	
High	358 (21.6)	−0.171 ± 0.879	
Health literacy (HLSAC)			
Low	158 (9.5)	0.318 ± 1.269	0.002
Average	1223 (73.5)	−0.020 ± 0.963	
High	282 (17.0)	−0.083 ± 0.962	

RB, risk behaviour standardised index; HLSAC, health literacy in school-aged children index; *p*, Mann–Whitney or Kruskal–Wallis depending on the number of groups being compared; M ± SD, mean ± standard deviation.

**Table 2:** Risk Behaviours undertaken in the last 30 days by adolescents aged 17 (%).

	Total	Boys	Girls	*p*
Smoking traditional cigarettes	32.5	31.3	33.5	0.340
Smoking e-cigarettes	28.7	34.6	23.6	<0.001
Alcohol use	73.7	72.8	74.4	0.479
Being drunk	34.9	37.9	32.3	0.018
Cannabis use	14.9	19.2	11.3	<0.001

**Table 3:** Spearman’s rank correlation between HLSAC and RBI indices by gender and type of school.

	General Secondary Schools			Vocational Secondary Schools		
	Total	Boys	Girls	Total	Boys	Girls
Spearman’s rho	−0.093	−0.047	−0.126	−0.078	−0.044	−0.126
*p*	0.005	0.367	0.003	0.038	0.399	0.023
*n*	901	367	534	701	376	325

RBI, risk behaviour standardised index; HLSAC, health literacy in school-aged children index.

**Table 4:** Estimation * of overall and type of school-specific linear models for standardised risk behaviours (RBI) index.

Independent Variables	Model 1-Total Group		Model 2a-General Secondary Schools		Model 2b-Vocational Secondary Schools	
	β	*p*	β	*p*	β	*p*
Constant		0.000		0.000		0.000
Gender	−0.122	0.000	−0.125	<0.001	−0.119	0.002
Place of living	−0.060	0.019	−0.070	0.042	−0.049	0.198
Type of school	0.040	0.144	-	-	-	-
School performance	−0.142	0.000	−0.130	<0.001	−0.158	<0.001
Family affluence (FAS)	0.078	0.002	0.067	0.046	0.085	0.024
Health literacy (HLSAC **)	−0.063	0.013	−0.075	0.026	−0.052	0.174
Quintiles of neighbourhood deprivation	−0.096	0.001	−0.062	0.072	−0.105	0.006
R-squared	0.066		0.053		0.064	
F test (*p*)	15.797 (<0.001)		8.162 (<0.001)		7.820 (<0.001)	

* Beta, standardised regression parameter. Gender: 1, male; 2, female. Type of school: 1, general education; 2, vocational. Place of living: 1, urban; 2, rural. Other variables are continuous. ** HLSAC - health literacy in school-aged children index.

**Table 5:** Estimation * of gender-specific linear models for standardised RB (RBI) index.

Independent Variables	Boys		Girls	
	Beta	*p*	Beta	*p*
Constant		0.157		0.000
Place of living	−0.009	0.804	−0.124	0.000
Type of school	0.048	0.252	0.026	0.484
School performance	−0.107	0.004	−0.195	0.000
Family affluence (FAS)	0.070	0.060	0.093	0.006
Health literacy (HLSAC)	−0.044	0.237	−0.084	0.015
Quintiles of neighbourhood deprivation	−0.067	0.116	−0.139	0.000
R-squared	0.028		0.089	
F test (*p*)	3.427 (0.002)		13.526 (<0.001)	

* Beta, standardised regression parameter. Type of school: 1, general education; 2, vocational. Place of living: 1, urban; 2, rural. Other variables are continuous.

## Data Availability

The data presented in this study are available on request from the HBSC network.

## Supplementary Materials

The following supporting information can be downloaded at: https://www.mdpi.com/article/10.3390/ijerph19159381/s1, Table S1: significance of factors in the general linear (GLM) models with and without interaction effect.

## Appendix A

**Table A1:** HLSAC index level according to sociodemographic characteristics in 17-year-olds.

	HLSAC Level (%)			*p*
	Low	Average	High	
Gender				
Male	11.1	71.1	17.8	0.055
Female	8.1	75.7	16.2	
Type of school				
General school (GE)	7.5	74.0	18.5	0.003
Vocational school (VT)	12.0	72.9	15.0	
Place of living				
Urban	8.8	71.5	19.7	0.002
Rural	10.4	76.4	13.2	
Quintiles of neighbourhood deprivation				
Q1—high deprivation	10.0	73.8	16.3	<0.001
Q2	13.7	74.6	11.7	
Q3	12.7	75.7	11.6	
Q4	9.0	73.7	17.3	
Q5—low deprivation	6.2	72.0	21.8	
Family affluence scale (FAS)				
Low	10.4	74.6	15.0	0.065
Average	9.2	74.7	16.1	
High	9.2	68.6	22.1	
School performance				
Low	16.0	70.1	13.9	<0.001
Average	7.5	77.3	15.1	
High	7.8	67.3	24.9	

HLSAC, health literacy in school-aged children index; p, chi-square test.
