# Supplementary material for: Who Is Able to Resist What Is Forbidden?—The Relationship between Health Literacy and Risk Behaviours in Secondary School Students in the Broader Social and Educational Context
Source: Int J Environ Res Public Health. 2022 Jul 31;19(15):9381. doi: 10.3390/ijerph19159381 (PMC9368140; doi:10.3390/ijerph19159381)
Supplement: Supplementary file 1 [file ijerph-19-09381-s001.zip › ijerph-1760022-supplementary.pdf]

Table S1. Significance of factors in the general linear (GLM) models with and without interaction effect.

	Model 1 -p	Model 2 - p
Factors		
1. Gender	0.000	0.000
2. School performance	0.000	0.105
3. Domicile	0.015	0.128
4. HLSAC categories	0.013	0.002
Covariates		
5. Local deprivation	0.000	0.000
6. Family Affluence (FAS)	0.004	0.006
3-way interaction		
2 * 3 * 4	-	0.017
R-sq adjusted	0.055	0.063

HLSAC - health literacy in school-aged children index.
